# Metabolic Pathway Genes Associated with Susceptibility Genes to Coronary Artery Disease

**DOI:** 10.1155/2018/9025841

**Published:** 2018-02-11

**Authors:** Heng Lu, Yi Chen, Linlin Li

**Affiliations:** ^1^Department of Cardiovascular Surgery, Union Hospital, Fujian Medical University, Fuzhou, China; ^2^Department of Nephrology, Union Hospital, Fujian Medical University, Fuzhou, China

## Abstract

Coronary artery disease (CAD) is one of the leading threats to global health. Previous research has proven that metabolic pathway disorders, such as high blood lipids and diabetes, are one of the risk factors that mostly cause CAD. However, the crosstalk between metabolic pathways and CAD was mostly studied on physiology processes by analyzing a single gene function. A canonical correlation analysis was used to identify the metabolic pathways, which were integrated as a unit to coexpress with CAD susceptibility genes, and to resolve additional metabolic factors that are related to CAD. Seven pathways, including citrate cycle, ubiquinone, terpenoid quinone biosynthesis, and N-glycan biosynthesis, were identified as an integrated unit coexpressed with CAD genes. These pathways could not be revealed as a coexpressed pathway through traditional methods as each single gene has weak correlation. Furthermore, sets of genes in these pathways were candidate markers for diagnosis and detection from patients' serum.

## 1. Introduction

Coronary artery disease (CAD) is a leading cause of global death with several risk factors, including smoking, hypertension, diabetes, obesity, high blood lipids, and stress [1–4]. The current risk assessment of CAD is based on the determination of these factors. Moreover, avoiding these risk factors is the most efficient means of preventing CAD [5, 6]. Generally, almost all risk factors of CAD are related to metabolism in views of basic biological research. For example, high blood lipids, which play an important role in causing CAD, is regulated by a complex metabolic network that involves the biosynthesis and degradation of cholesterol, triglycerides, and lipoproteins [7–10]. Another risk factor, diabetes, is a metabolic disease related to glucose [11, 12]. The relationship between diet and risk of CAD also suggests that the metabolic pathway plays a pivotal role in the pathophysiology of CAD. Recent studies revealed that vegetarians as well as Mediterranean diet people have less cases of CAD [13–17]. In general, pathophysiology and dietetics research have determined the close link between metabolism and CAD.

In another side, molecular genetics research also accumulated several evidences which suggest that genetic factors contribute lots to the susceptibility of CAD. Although, in comprehensive studies, susceptible genes to CAD have been revealed to be involved in diverse biological pathways such as inflammation, innate immunity, and cholesterol metabolism. However, genome-wide crosstalks between susceptibility genes and metabolism pathways still remained to be characterized.

To understand the crosstalk between genes in the genome level, the transcriptome network is an efficient way to mining important interaction. The most common model for transcriptome network analysis is coexpression which calculates the correlation between genes in certain conditions. However, the traditional approach is only suitable for a single gene pair. So far, limited transcriptome network analysis works with integrated pathways which is consisted of many genes [18]. Canonical correlation analysis (CCA) is a powerful method to measure the coexpression between two sets of genes [19]. To detect the genetic regulatory variants on the Childhood Asthma Management Program, researchers used CCA to successfully detect candidate genes. The genes related to the glioma pathway were also identified by CCA from the transcriptome of glioblastoma in patients. The transcriptome plays a key role in studies on AIDS restriction genes using the CCA-determined purine metabolism pathway [20].

In this study, CCA was used to identify the coexpression between integrated metabolic pathways and CAD genes. The most significant metabolic pathways that could be a potential candidate disease marker for CAD diagnosis are discussed.

## 2. Materials and Methods

### 2.1. Datasets

Human genome expression datasets were downloaded from the website of COPRESDB (http://coxpresdb.jp/), which contains approximately 4000 experiments and expression data on 20,000 human genes. Metabolic pathway genes were downloaded from KEGG (http://www.kegg.jp), which includes 129 typical metabolic pathways with predicted genes. The CAD genes were collected from published literature. Two expression datasets were generated to include the metabolic pathway gene and CAD expression data.

### 2.2. CCA

CCA was performed to dissect the correlations between CAD and metabolic pathway gene expression. In concept, CCA could integrate multiple correlations into an abstract group. The correlation level between two sets of genes can be determined by scoring the relationship between the groups. In statistics, this method generates independent pairs of new variables from the original two sets of variables, namely, the canonical variable. CCA is a method processing cross-covariance matrices. If there are correlations among the variables from two vectors **X** = (*X*_1_,…, *X*_*n*_) and **Y** = (*Y*_1_,…, *Y*_*m*_) of random variables, CCA could detect linear combination of the *X_i_* and *Y_j_* with maximum correlation. As a result, the canonical variable is a linear combination of the original variables.

Vector **A**_*i*_ = (*a*_1_, *a*_2_, *a*_3_,…, *a*_*n*_) describes the CAD gene *i* with different expression levels from different experiments (1 to *n*). Vector **B**_*i*_ = (*b*_1_, *b*_2_, *b*_3_,…, *b*_*n*_) describes the metabolic gene *i* with different expression levels from different experiments (1 to *n*). Matrix **M** = (*A*_1_, *A*_2_, *A*_3_,…, *A*_*n*_) is for CAD genes, and matrix **N**_*i*_ = (*B*_1_, *B*_2_, *B*_3_,…, *B*_*n*_) is for a certain metabolic pathway *i*. CCA were calculated between matrix **M** and each **N** *_i_* by following the steps in the *R* program:

1. *zscore (M) and zscore (N_i_);*
2. *r_11_ = corr (zscore (M)), r_22_ = corr (zscore(N_i_));*
3. *[AA, BB, r, U, V, stats] = canoncorr (zscore (M), zscore (N_i_));*
4. *r_1A_ = AA'^∗^r_11_, r_2B_ = BB'^∗^r_22_;*
5. *S_a_ = sum (r_1A_.^2/length (r_1A_), S_b_ = sum (r_2B_ ^2/length (r_2B_));*
6. *Screen the candidate CCA using a certain threshold for r P value, S_a_, and S_b_. (in this study, the thresholds were 0.6, 0.00001, 0.3, and 0.15)*

*S_a_* and *S_b_* were used to validate the percentage of genes in total CADs or a certain metabolic pathway, which could be presented by canonical variables. A high level of *S_a_* or *S_b_* indicates that more genes belonging to the group are involved in the correlation. Thus, not only the correlation between gene pairs was identified but also the correlation between sets.

### 2.3. Software Tools

CCA was performed using the *R* platform (http://www.r-project.org/). The web-based DAVID tool (http://david.abcc.ncifcrf.gov/) was used for gene ontology enrichment analysis for the metabolic pathway. The STRING platform was used for the contraction of the PPI network.

### 2.4. Sample Collection

Fifty serum samples from Chinese patients (Union Hospital of Fujian Medical University) were collected and divided into two groups, namely, CAD (from CAD patients) and control (from healthy people). The blood samples were maintained at 37°C for 1 h and centrifuged at 3000 g/min for 20 min at 4°C, and the supernatant fluid is collected at −80°C until testing. All clinical research experiments were approved by the Medical Ethics Committee of Union Hospital of Fujian Medical University and performed according to the approved guidelines.

### 2.5. RNA Isolation, cDNA Synthesis, and q-PCR Analysis

The total RNA was extracted using TRIzol reagent (Life Technologies), following the manufacturer's instructions with slight modifications. Up to 1 *μ*g of the total RNA was used by utilizing the First Strand cDNA Synthesis Kit (TOYOBO) to generate cDNA according to the manufacturer's instructions. q-PCR was performed using the TransStart Top Green q-PCR SuperMix (TransGen Biotech) and was conducted using the iQTM5 multicolor real-time PCR detection system (Bio-Rad).

## 3. Results and Discussion

### 3.1. Strategy

**Figure 1:**
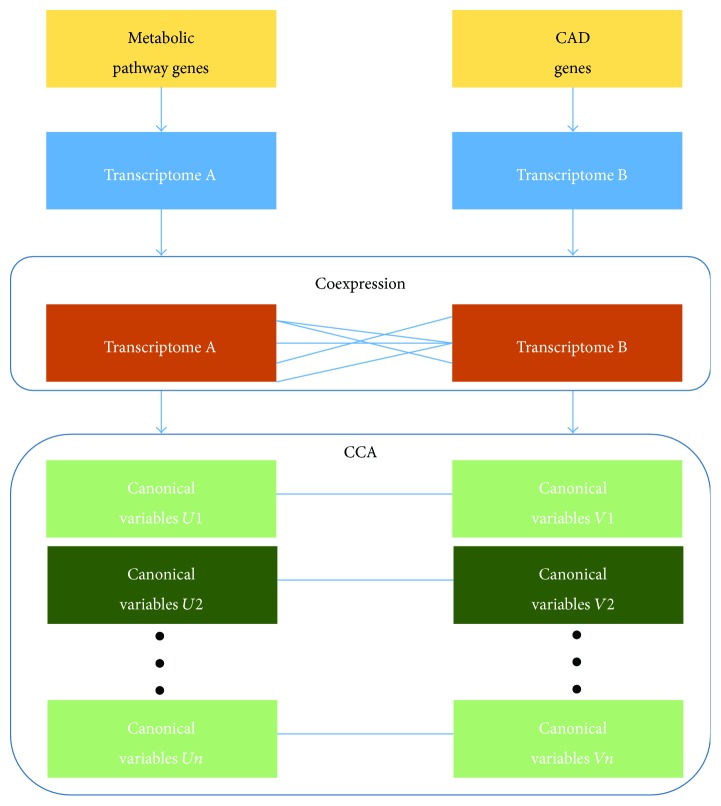
Strategy of study for the canonical correlation analysis. Collection of CAD genes and metabolic pathway genes; construction of the coexpression network among individual CAD and metabolic pathway genes; performance of CCA; and analysis of canonical variables.

In this study, a clustering for the expression pattern of CAD and metabolic pathway genes was first created to estimate the possible gene group, which could provide an evidence for identifying the correlation among gene groups. Next, a coexpression network was constructed among individual CAD and metabolic pathway genes. This step is prepared for comparing with CCA. Then, CCA was performed to identify the canonical variables that delegate the integrated CAD and metabolic pathway genes (Figure 1). In particular, additional genes in a pathway could be regulated by the same factor. Thus, the whole pathway was suggested to be controlled by the same factor. In this study, an entire metabolic pathway was correlated with the entire CAD genes.

### 3.2. CAD and Metabolic Pathway Genes

A published literature shows that SNPs associated with coronary heart disease and mean arterial pressure were collected for further analysis. There are Mendelian forms of CAD that show mutations in genes that are related to low- and high-density lipoprotein metabolism or homeostasis. The heritability of fatal coronary events is estimated at 57% and 38% in monozygotic and dizygotic twin cohorts, respectively. Researchers have long recognized the familial clustering of CAD. The first linkage of premature CAD to loci on chromosomes 2q21.2-22 and Xq23-26 was found in Finland [21]. Subsequently, several studies from Germany, the United States, and England identified a novel chromosome locus linked with CAD [22]. Different genes, such as ALOX5AP (5-lipoxygenase-activating protein), are characterized by haplotypes that are associated with CAD. Several genome-wide association studies promoted by the improved genome sequence technology are performed on CAD. A novel replicated locus on chromosome 17 was found by genome-wide mapping of susceptibility to CAD [23]. In Indo-Mauritians, a susceptibility locus on chromosome 16p13 is revealed by a genome-wide scan for CAD [24]. Recently, a new locus on chromosome 10p11.23 for CAD is discovered by a genome-wide association study [25]. In addition, a genome-wide linkage analysis for CAD and its related risk factors is conducted [26].

Eight studies identified 373 SNPs associated with coronary heart disease-related phenotype at a 10*E*5 *P* level. These SNPs are located at several chromosomes inside coding or noncoding regions. In general, the coding regions match 120 genes involved in diverse biological processes, such as response to a stimulus, developmental, rhythmic, metabolic, and immune system processes (Figure S1, Table S1). Moreover, the expression of CAD-associated genes was controlled during the progress of the disease. Thus, the expression levels of 128 genes were collected from human transcriptome database with approximately 5000 experiments.

Then, genes that are related to metabolic pathways were collected from the KEGG database. In general, 84 metabolic pathways, including metabolism of carbohydrate, energy, lipid, nucleotide, amino acid, glycan biosynthesis, cofactors and vitamins, terpenoids and polyketides, and biosyntheses of other secondary metabolites (Table S2), cover major metabolic processes in human biology. A total of 300 gene code putative enzymes exist for these pathways according to the current knowledge on the human genome. Currently, certain metabolic pathways, such as D-arginine and D-ornithine metabolism, valine, leucine and isoleucine biosyntheses, lysine biosynthesis, and biotin and lipoic acid metabolism, contain only 1–3 genes. These pathways have a limited value for further CCA. A total of 77 pathways, such as glycolysis/gluconeogenesis, TCA cycle, and pentose phosphate pathway (Table S2), have more than five genes in the human genome. These pathways could be subject to CCA because we aim to estimate the correlation between the integrated CAD gene group and metabolic gene pathway, not the individual gene.

### 3.3. Coexpression and the PPI Network

There are huge transcriptome data related to CAD carried out by microarray and next-generation sequencing. For example, researchers establish a mouse heart transcriptomic network sensitivity to various heart diseases using microarray [27]. Cardiac hypertrophy was also studied by transcriptome microarrays to identify long noncoding RNAs [28]. A gene network was established in low-dose, radiation-affected cardiomyocytes [29]. Many transcriptional regulators in human ischemic cardiomyopathy are revealed from the gene expression network [30]. Transcriptome sequencing of different cell lines identifies several candidate genes related to coronary artery calcification [31]. Some potential candidate genes for heart failure were also discovered by transcriptome network analysis [32]. Transcriptome network analysis is also used to find divergent cardiovascular disease pathways between animal and humans [33].

**Figure 2:**
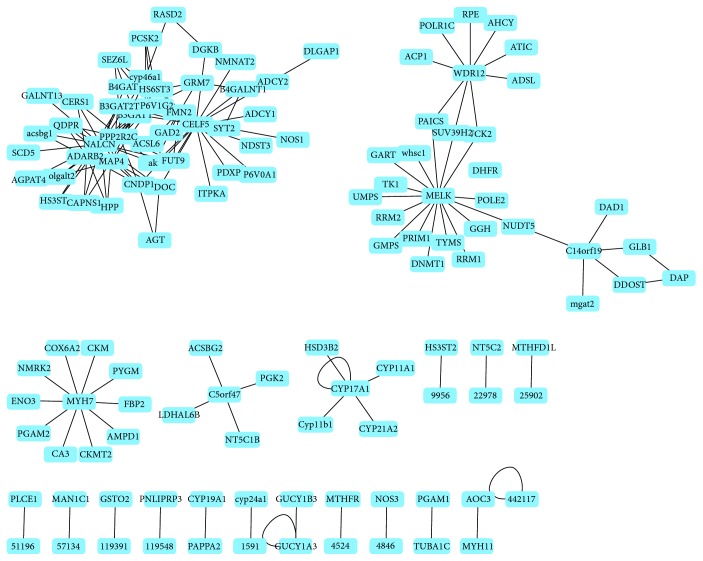
Coexpression network of CAD genes and metabolic pathway genes. The connection indicates the Pearson correlation factor *r* > 0.6.

A coexpression network was constructed using the Pearson correlation to determine the relationship between all CAD susceptibility and metabolic pathway genes. Numerous genes have connections with high *r* value (*r* > 0.6). A total of 24 CAD genes, namely, *105* (*ADARB2*), *183* (*AGT*), *1586* (*CYP17A1*), *1611* (*DAP*), *2917* (*GRM7*), *2982* (*GUCY1A3*), *4134* (*MAP4*), *4625* (*MYH7*), *4629* (*MYH11*), *5126* (*PCSK2*), *5522* (*PPP2R2C*), *9229* (*DLGAP1*), *9833* (*MELK*), *23544* (*SEZ6L*), *23551* (*RASD2*), *55017* (*C14orf119*), *55759* (*WDR12*), *56776* (*FMN2*), *60676* (*PAPPA2*), *60680* (*CELF5*), *84790* (*TUBA1C*), *127833* (*SYT2*), *133491* (*C5orf47*), and *259232* (*NALCN*), were identified in this coexpression network (Figure 2, Table S3). The network clearly shows several disconnected subnetworks. Six subnetworks, namely, 9833 (maternal embryonic leucine zipper kinase (MELK)), 55759 (WD repeat domain 12 (WDR12)), 55017 (C14orf119), 4625 (myosin, heavy chain 7, cardiac muscle (MYH7)), 133491 (C5orf47), and 1586 (cytochrome P450, family 17, subfamily A, polypeptide 1 (CYP17A1)) (Figure 2), have different genes at the center. In addition, one subnetwork has multiple genes at the center.

In this coexpression network, CAD genes were connected to 80 metabolic pathway genes (Table S4). CAD genes were coexpressed with the metabolic pathway. These metabolic pathway genes were enriched in a diverse metabolic pathway, such as purine metabolism, biosynthesis of antibiotics, pyrimidine metabolism, and glycolysis/gluconeogenesis (*P* value < 10*E* − 03). Furthermore, several metabolic pathways with less than four genes coexpressed with CAD genes. For example, 23205, 81616, and 23305 belonging to fatty acid biosynthesis exist in this network. The coexpression network between CAD and metabolic pathway genes could not identify the connection between the integrated pathways but simply link from point to point.

**Figure 3:**
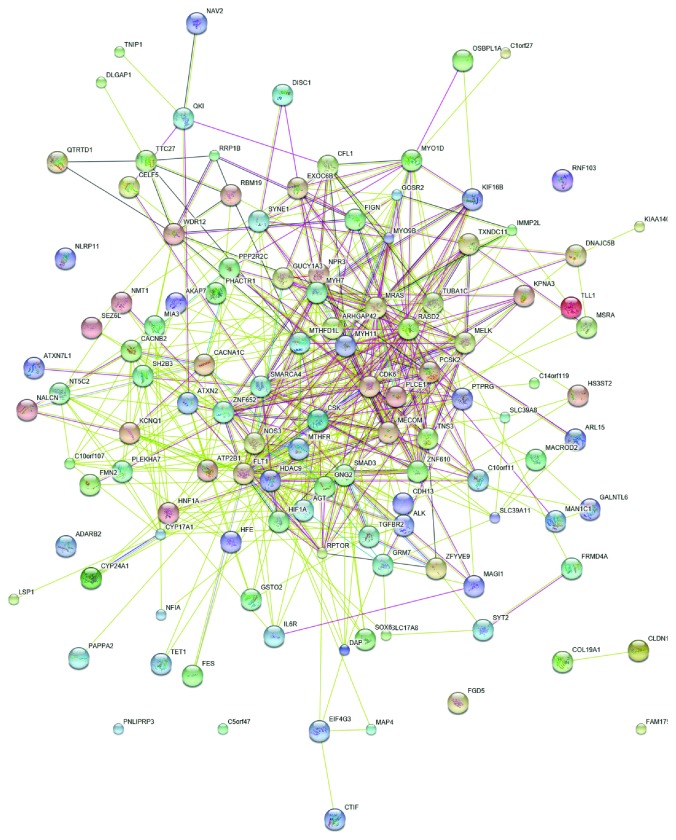
PPI network of CAD genes. Note that no metabolic pathway was enriched in the PPI network.

Furthermore, a PPI network was constructed using CAD genes as baits with low constraints by the STRING platform (Figure 3). Several interactions between CAD genes were observed. Moreover, many interaction proteins were identified through CAD baits. However, the KEGG enrichment analysis showed that all these genes only were enriched in adrenergic signaling in cardiomyocytes, tight junction, and glutamatergic synapse. No metabolic pathway was found while enriched KEGG is processed in the PPI network.

### 3.4. CCA of CAD Susceptibility and Metabolic Pathway Genes

**Figure 4:**
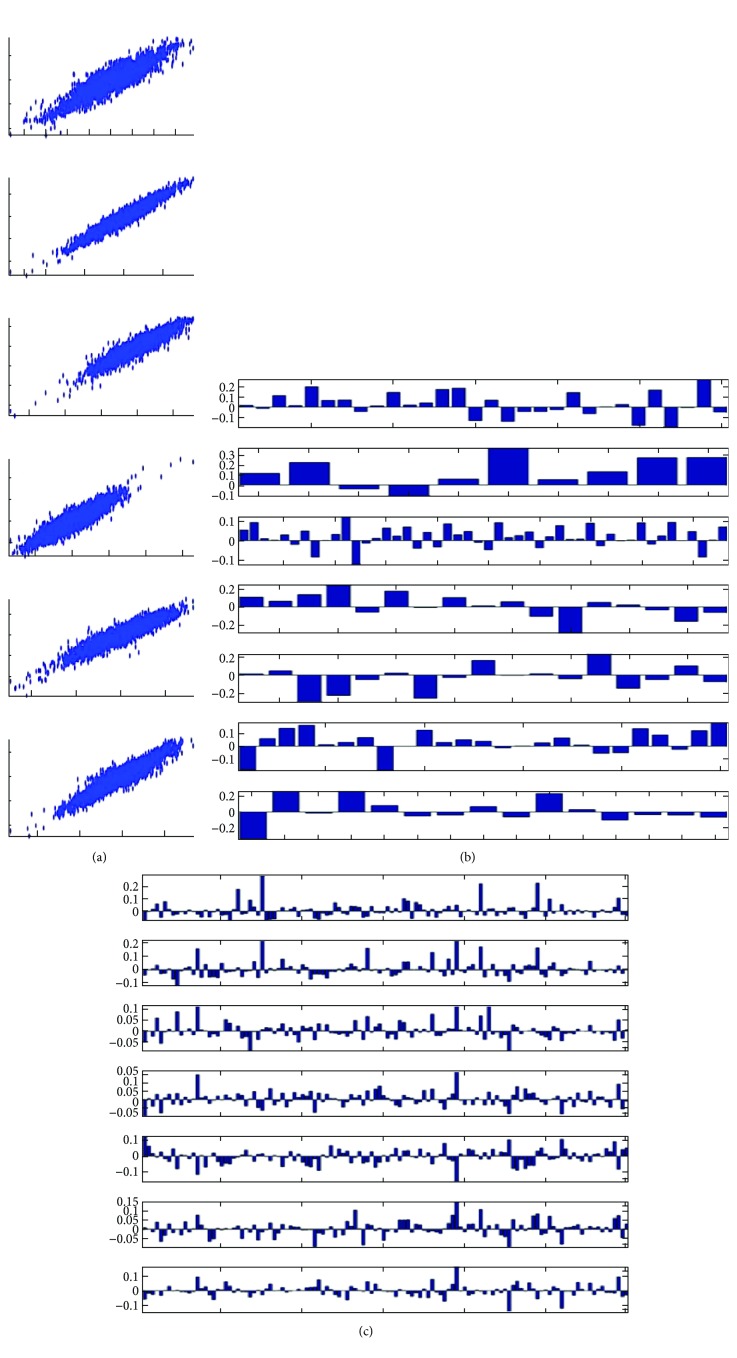
Characteristic of canonical variables indicating integrate pathway. A threshold was set to identify the CCA which cover whole pathway (*S_a_*, *S_b_* > 0.2, *r* values > 0.5, and *P* values < 0.001). (a) Scatter plots of seven CCA pairs with top *r* values. Note that all identified CCA were highly correlated. (b) The coefficient of metabolic pathway genes on seven canonical variables. Each metabolic pathway contains different number of genes. The genes with big values contributed more in CCA. (c) The coefficient of CAD genes on seven canonical variables. The genes with big values contributed more in CCA.

CCA was used to resolve canonical variables, correlation coefficient, and standard deviation to determine the significant canonical correlations between CAD and metabolic pathway genes in the transcriptome. The canonical variables are a new set of statistically independent variable pairs generated from the original variables, which describe the level of gene expression in different experiments. The canonical variables, which are independent, could be linearly combined to create original variables and could be considered as a component of the original variables. We set a standard to evaluate the canonical variables, which could delegate an integrated pathway at a maximum. Thus, we isolated the canonical variables with large *S_a_*, *S_b_* (>0.2), *r* values (>0.5), and minimal *P* values (<0.001). The standard deviations indicated the extent of coverage of canonical variables over the entire pathway. Thus, we selected metabolic pathways with more than 10 genes in the genome for further analysis to identify the correlation among the integrated pathways or among single genes.  Table 1 indicated that only seven sets of canonical variables that satisfy all our requirements were detected. These variables are related to seven metabolic pathways, including TCA cycle, ubiquinone and other terpenoid quinone biosyntheses, N-glycan biosynthesis, other glycan degradation, glycosaminoglycan degradation, and glycosylphosphatidylinositol (GPI) anchor biosynthesis and glycosphingolipid biosynthesis–ganglioseries. None of these metabolic pathways were identified by the Pearson correlation or PPI networks. The correlation coefficient factors were more than 0.9 (Figure 4(a)). *S_a_* indicated that the standard deviation for metabolic pathways had a maximum of 0.3 Table 1. *S_b_* indicated that the standard deviation of CAD genes varies from 0.19 to 0.93 Table 1. The results suggested that the integrated coexpression in metabolic pathways is not at high level. However, CAD genes were highly integrated to coexpress with metabolic pathways. *S_b_* was equal to 0.93 for coexpression between CAD genes and ubiquinone and other terpenoid quinone biosyntheses, indicating that 93% of CAD genes can be delegated by canonical variables (Figure 4(a)–4(c)). The results were discussed with published literature by showing the interaction between metabolic pathways and CAD genes.

### 3.5. TCA Cycle

Table 1 indicates that the canonical variable represents 20% of the variability in the original expression pattern of TCA cycle pathway genes and 19% of the variability in the expression pattern of CAD genes. The top 6 genes with big absolute value of coefficient factor is selected as representative genes for this canonical variable (Figure 4(b), Table 1). These genes were *8803* (*SUCLA2*), *1737* (*DLAT*), *8801* (*SUCLG2*), *4191* (*MDH2*), *6392* (*SDHD*), and *4190* (*MDH1*).

In the last decades, several studies provide evidence that supports the interaction between CAD and TCA cycle. For example, the degradation of myocardial aspartate and glutamate is induced by the complete citric acid cycle, ischemia, and hypoxia. This process resulted in the production of succinate in isolated hearts [34]. Also, it has been revealed the ATP production in the absence of oxygen through this channeling which could provide cardioprotection [35]. In another case, the accumulation of TCA cycle intermediate fumarate is beneficial for stabilizing hypoxia-inducible factor 1*α* (HIF-1*α*), which is a key transcription factor that controls the response to hypoxia and myocardial protection [36]. Together, it is proposed that manipulation of the citrate cycle could increase cardioprotection.

### 3.6. Ubiquinone and Other Terpenoid Quinone Biosyntheses

Table 1 presents that the canonical variable comprises 30% of the variability in the original expression pattern of ubiquinone and other terpenoid quinone biosynthesis pathway genes and 93% of the variability in the expression pattern of CAD genes. The top three genes with a high absolute value of coefficient factor, namely, *27235* (*COQ2*), *84274* (*COQ5*), and *79001* (*vkorc1*), are selected to represent the genes for this canonical variable (Figure 4(b), Table 1).

The key role of ubiquinone in CAD has been accepted by several researchers extensively. Coenzyme Q10, which is a kind of ubiquinone, is supplemented to improve diastolic heart functions, especially in CAD patients. Moreover, it is the major method of treating current diseases [37]. Coenzyme Q10, which could reduce oxidative stress and induce antioxidant activity in CAD patients, is the proposed mechanism [38]. This method is helpful for low-density lipoprotein cholesterol and vitamin E. In particular, this mitochondrial coenzyme is a vital factor for producing ATP [39]. These functions of coenzyme Q10 in antioxidant and energy production imply its role in coronary revascularization. The research demonstrates a significant correlation between the risk of CAD and levels of coenzyme Q10 [40]. Many patients with cardiovascular diseases, such as CAD, cardiomyopathy, congestive heart failure, angina pectoris, and hypertension, showed a deficiency in coenzyme Q10. The deficiency of coenzyme Q10 is a risk factor for cardiovascular disorder, especially increasing the early mortality in myalgic encephalomyelitis/chronic fatigue syndrome [41]. Current studies have proposed that low plasma coenzyme Q10 should be considered a risk factor for CAD and a marker for chronic fatigue in depression [42]. Coenzyme Q10 has been regarded as an independent factor of mortality in congestive heart failure. Furthermore, coenzyme Q10 improves the immune system in vertigo and Meniere' disease-like syndrome [43]. In general, coenzyme Q10 is significant in clinical therapy and diagnosis of CAD patients.

### 3.7. Glycosaminoglycan Degradation

As shown in Table 1, the canonical variable represents 22% of the variability in the original expression pattern of glycosaminoglycan degradation pathway genes and 69% of the variability in the expression pattern of CAD genes. The top four genes with a high absolute value of coefficient factor, namely, *2720* (*GLB1*), *3074* (*HEXB*), *6677* (*SPAM1*), and *2799* (*GNS*), are selected to represent the genes for this canonical variable (Figure 4(b), Table 1).

Studies on the link between glycosaminoglycan and CAD were limited in the last decades. However, the clues were already revealed by research on certain kinds of glycosaminoglycan with antioxidative activity. A pilot study has been conducted with sulodexide, a glycosaminoglycan that primarily consists of heparin, which mainly causes oxidative stress [44]. Sulodexide treatment could decrease only the level of plasma 8-isoprostane but not LDL cholesterol, triglycerides, fibrinogen, and C-reactive protein. However, a significant reduction in oxidative stress in CAD patients administered with sulodexide was observed, implying the important role of plasma 8-isoprostane and glycosaminoglycan. A study showed that another long-chain glycosaminoglycan*-*hyaluronan could interact with versican to create matrices, which are required for arterial smooth muscle growth during the vascular disease [45]. The histopathological studies on coronary arteries revealed that abundant glycosaminoglycan is associated with percutaneous transluminal coronary angioplasty [46].

### 3.8. Glycosphingolipid Biosynthesis–Ganglioseries

In Table 1, the canonical variable accounts for 22% of the variability in the original expression pattern of glycosphingolipid biosynthesis–ganglioseries pathway genes and 92% of the variability in the expression pattern of CAD genes. The top three genes with a high absolute value of coefficient factor, namely, 2583 (*B4GALNT1*), 2720 (*GLB1)*, and 3074 (*HEXB*), are selected to represent genes for this canonical variable (Figure 4(b), Table 1).

The role of glycosphingolipid biosynthesis–ganglioseries in CAD disease was also revealed by recent studies. Glycosphingolipids act as messengers that are integrated into the cell membrane to transduce the growth factor. High levels of cholesterol in the blood are proven to be associated with glycosphingolipid. Recent studies concluded that glycosphingolipids were involved in the major lipoprotein classes in normal and dyslipoproteinemic sera [47]. Cholesterol efflux could be inhibited by the accumulation of glycosphingolipid [48]. The cellular cholesterol homeostasis is induced by glycosphingolipid storage [49]. Studies found that arterial stiffness and atherosclerosis could be ameliorated by inhibiting glycosphingolipid synthesis in mice and rabbit models given the pivotal role of cholesterol in CAD [50].

### 3.9. Other Pathways without Literature

In Table 2, the canonical variable comprises 23% of the variability in the original expression pattern of N-glycan biosynthesis pathway genes and 28% of the variability in the expression pattern of CAD genes. The top 11 genes with a high absolute value of coefficient factor, namely, *4247* (*mgat2*), *4248* (*MGAT3*), *85365* (*ALG2*), *1650* (*DDOST*), *11282* (*MGAT4B*), *79868* (*ALG13*), *57134* (*MAN1C1*), *8813* (*DPM1*), *4122* (*MAN2A2*), *146664* (*MGAT5B*), and 29929 (*ALG6*), are selected to represent the genes for this canonical variable (Figure 4(b), Table 1).

As shown in Table 1, the canonical variable explains 27% of the variability in the original expression pattern of other glycan degradation pathway genes and 62% of the variability in the expression pattern of CAD genes. The top five genes with a high absolute value of coefficient factor, namely, *10825* (*NEU3*), *2720* (*GLB1*), *074* (*HEXB*), *129807* (*NEU4*), and 2519 (*FUCA2*), are selected to represent the genes for this canonical variable (Figure 4(b), Table 1).

Table 1 also shows that the canonical variable represents 21% of the variability in the original expression pattern of the GPI anchor biosynthesis pathway genes and 43% of the variability in the expression pattern of CAD genes. The top five genes with a high absolute value of coefficient factor, namely, (*PIGQ*), *284098* (*PIGW*), *2822* (*GPLD1*), *5281* (*PIGF*), and 5279 (*PIGC*), are selected to represent the genes for this canonical variable (Figure 4(b), Table 1).

### 3.10. Validation of Expression Genes as Candidate Disease Markers for CAD

**Figure 5:**
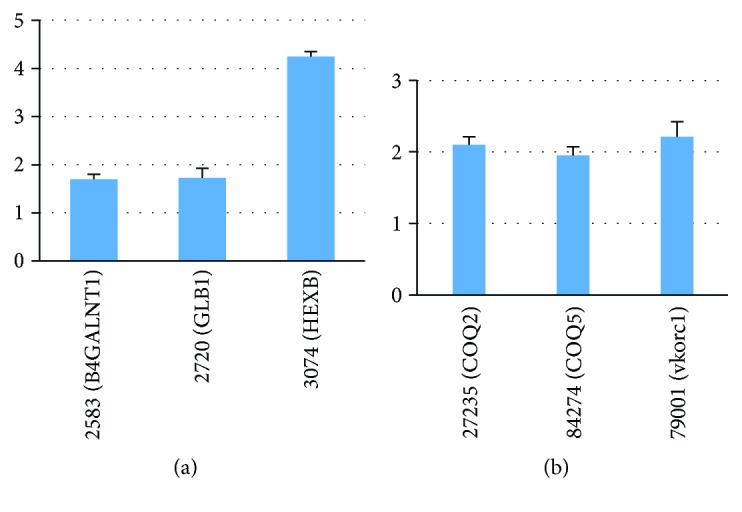
qRT-PCR of metabolic pathway genes in CAD patients. (a) *2583* (*B4GALNT1*), *2720* (*GLB1*), and *3074* (*HEXB*) in glycosphingolipid biosynthesis. (b) *27235* (*COQ2*), *84274* (*COQ5*), and *79001* (*vkorc1*) in ubiquinone and other terpenoid quinone biosyntheses.

To explore the potential application of these metabolic pathway genes as disease markers for CAD, we used q-PCR for analyzing the expression pattern of CAD genes and its canonical correlated metabolic pathways. As more than 90% of CAD genes are associated with ubiquinone and other terpenoid quinone biosyntheses and glycosphingolipid biosyntheses–ganglioseries, we selected genes from both metabolic pathways for further analysis. Fifty serum samples were collected from the CAD patients and the control group. The expression levels of metabolic genes, namely, *2583* (*B4GALNT1*), *2720* (*GLB1*), and *3074* (*HEXB*), were determined by q-PCR. Except *3074* (*HEXB*) with 4 times induction, *2583* (*B4GALNT1*) and *2720* (*GLB1*) only increased 1.7 times in CAD patients (Figure 5(a)). The metabolic genes showed significant difference between CAD patients and the control group with a *P* value of less than 10*E* − 4. Six metabolic genes showed a significant difference between the CAD patients and the control group with a *P* value of less than 10*E* – 4. In traditional method, it is difficult to justify these genes as disease markers with less than two times induction. However, three genes together identified from CCA showed a significant *P* value. Thus, it suggests that a combination of genes is a potential disease marker for CAD by checking the expression levels rather than single genes. Also, genes *27235* (*COQ2*), *84274 (COQ5*), and *79001* (*vkorc1*) in ubiquinone and other terpenoid quinone biosynthesis pathways were also determined by qRT-PCR in these samples. A similar result was found as the glycosphingolipid biosynthesis pathway. Each gene was induced only 2 times in CAD patients (Figure 5(b)). However, three genes together showed a very significant *P* value. In statistics, the CCA method indicated that this combination of genes could be potential disease markers.

## 4. Conclusion

In this study, the canonical correlation between CAD and metabolic pathways gene expressions was analyzed. The results showed that the TCA cycle, ubiquinone and other terpenoid quinone biosyntheses, N-glycan biosynthesis, other glycan degradation, glycosaminoglycan degradation, GPI anchor biosynthesis, and glycosphingolipid biosynthesis–ganglioseries are the most significant metabolic pathways correlated with CAD genes. Furthermore, these metabolic pathway genes are beneficial for the diagnosis and detection of CAD through the quantification of the expression level of patients' serum.

## Figures and Tables

**Table 1:** Enriched metabolic pathway in the coexpression network.

Term	Count	Pop	*P* value	Genes
hsa00230: purine metabolism	17	176	8.46*E* − 11	5427, 9533, 2618, 6240, 5557, 11164, 6241, 26289, 270, 158, 93034, 8833, 10606, 471, 108, 107, 2983
hsa01130: biosynthesis of antibiotics	14	212	6.52*E* − 07	6120, 5224, 5232, 5223, 230, 2618, 26289, 270, 158, 8789, 92483, 10606, 471, 2027
hsa00240: pyrimidine metabolism	10	104	2.40*E* − 06	93,034, 5427, 9533, 7372, 7371, 6240, 5557, 7298, 6241, 7083
hsa00010: glycolysis/gluconeogenesis	7	67	1.06*E* − 04	92483, 5224, 5232, 5223, 230, 2027, 8789
hsa00670: one carbon pool by folate	4	20	0.001425311	1719, 471, 2618, 7298
hsa01230: biosynthesis of amino acids	6	74	0.001495954	6120, 5224, 5232, 5223, 230, 2027
hsa01200: carbon metabolism	7	113	0.001764573	6120, 5224, 5232, 5223, 230, 2027, 8789
hsa04913: ovarian steroidogenesis	5	49	0.002269906	1583, 108, 107, 3284, 1588
hsa00330: arginine and proline metabolism	5	50	0.002446109	84735, 1158, 4842, 57571, 1160
hsa00140: steroid hormone biosynthesis	5	58	0.004206383	1583, 1589, 3284, 1588, 1584
hsa00410: beta-alanine metabolism	4	31	0.005135035	84735, 2572, 57571, 8639
hsa00514: other types of O-glycan biosyntheses	4	31	0.005135035	10690, 135152, 27087, 23127
hsa00061: fatty acid biosynthesis	3	13	0.00927769	23205, 81616, 23305
hsa00790: folate biosynthesis	3	14	0.010744279	5860, 1719, 8836
hsa04925: aldosterone synthesis and secretion	5	81	0.013544109	1583, 108, 107, 1589, 3284
hsa00983: drug metabolism—other enzymes	4	46	0.015345429	8833, 7372, 7371, 7083
hsa01212: fatty acid metabolism	4	48	0.017202919	23205, 79966, 81616, 23305
hsa04922: glucagon signaling pathway	5	99	0.026291005	92483, 5224, 5223, 5837, 108
hsa00534: glycosaminoglycan biosynthesis—heparan sulfate/heparin	3	24	0.030276378	222537, 9348, 266722
hsa03320: PPAR signaling pathway	4	67	0.040871389	23205, 79966, 81616, 23305
hsa00030: pentose phosphate pathway	3	29	0.042939357	6120, 230, 8789
hsa00760: nicotinate and nicotinamide metabolism	3	29	0.042939357	93034, 27231, 23057

**Table 2:** The canonical variable delegate integrates metabolic pathways.

Metabolic pathways	Gene number	*r*	*S_a_*	*S_b_*	Represent gene ID
Citrate cycle (TCA cycle)	30	0.90	0.20	0.19	8803^∗^, 1737^∗^, 8801^∗^, 4191^∗^, 6392^∗^, 4190^∗^
Ubiquinone and other terpenoid quinone biosyntheses	10	0.90	0.30	0.93	27235, 84274, 79001
N-glycan biosynthesis	48	0.96	0.23	0.28	4247, 4248, 85365, 1650, 11282, 79868, 57134, 8813, 4122, 146664, 29929
Other glycan degradation	17	0.92	0.27	0.62	10825, 2720, 3074, 129807, 2519
Glycosaminoglycan degradation	17	0.91	0.22	0.69	2720, 3074, 6677, 2799
Glycosylphosphatidylinositol (GPI) anchor biosynthesis	25	0.93	0.21	0.43	9091, 284098, 2822, 5281, 5279
Glycosphingolipid biosynthesis–ganglioseries	15	0.93	0.22	0.92	2583, 2720, 3074

*S_a_* and *S_b_* indicate the extent of canonical variables covering the whole pathway. The representative genes were selected based on the percentage of covering (^∗^gene number in pathways *S_b_*).

## Supplementary Materials

Supplementary 1 Figure S1: functional category of CAD genes.

Supplementary 2 Table S1: list of CAD genes in this study. Table S2: list of metabolic pathways used in this study. Gene number indicated totally predicted genes in the pathways. Table S3: CAD gene ID identified in the coexpression network. Table S4: metabolic gene ID identified in the coexpression network.
