# Supplementary figures and images for: Metabolic Pathway Genes Associated with Susceptibility Genes to Coronary Artery Disease
Source: Int J Genomics. 2018 Feb 11;2018:9025841. doi: 10.1155/2018/9025841 (PMC5828413; doi:10.1155/2018/9025841)

## Slide 1
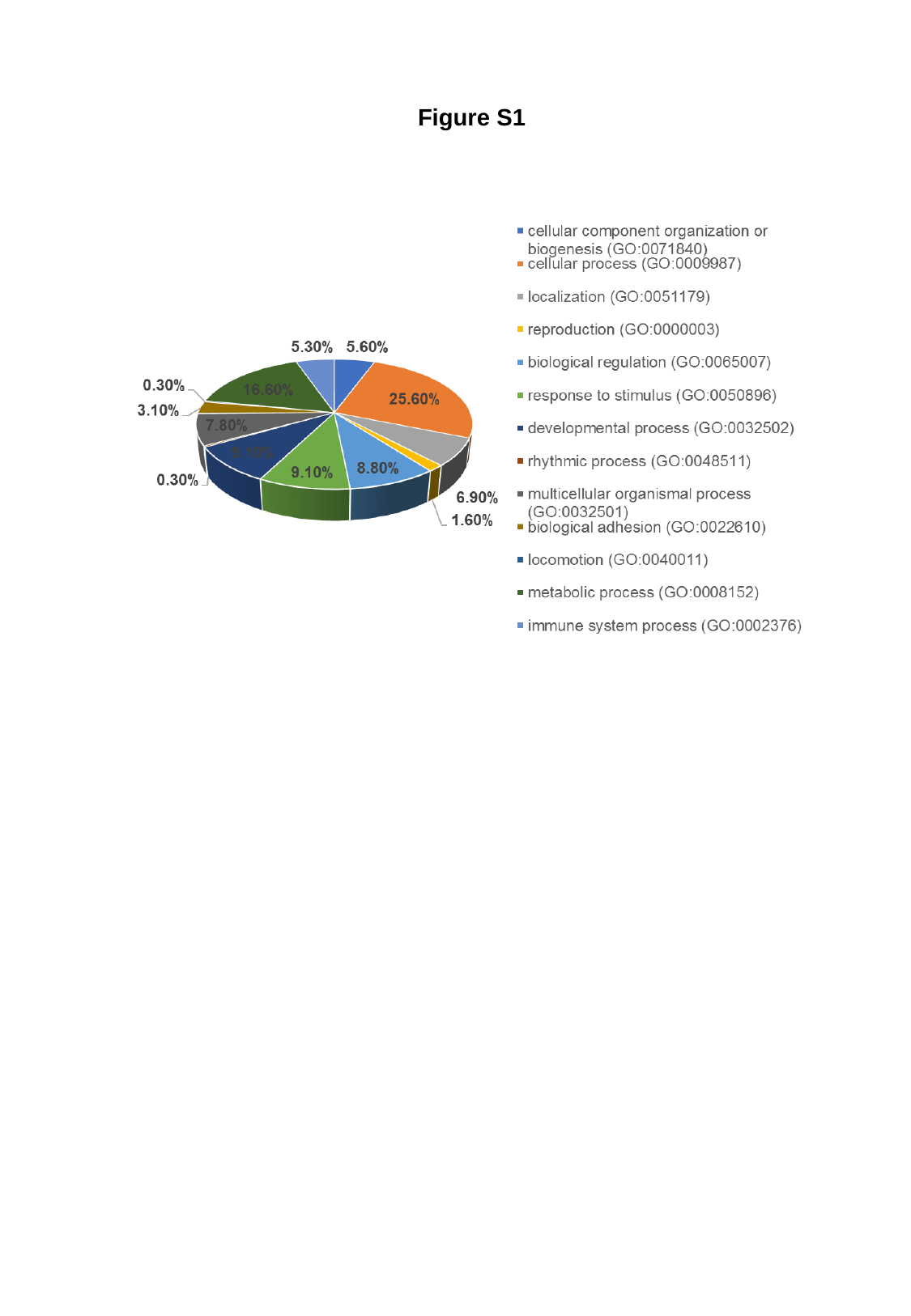

Figure S1

Supplement: Supplementary 1 — Figure S1: functional category of CAD genes. [file 9025841.f1.pptx]
